# Progressed Ulcerative Chest Wall Mass: A Case of Delayed Diagnosis and Intervention

**DOI:** 10.1016/j.acepjo.2025.100303

**Published:** 2025-12-23

**Authors:** Shreya Suresh, Eric Shipley, Jou-Tzu (Jennifer) Chen

**Affiliations:** Emergency Department, Overlake Medical Center & Clinics, Bellevue, Washington, USA

## Patient Presentation

**Figure 1:**
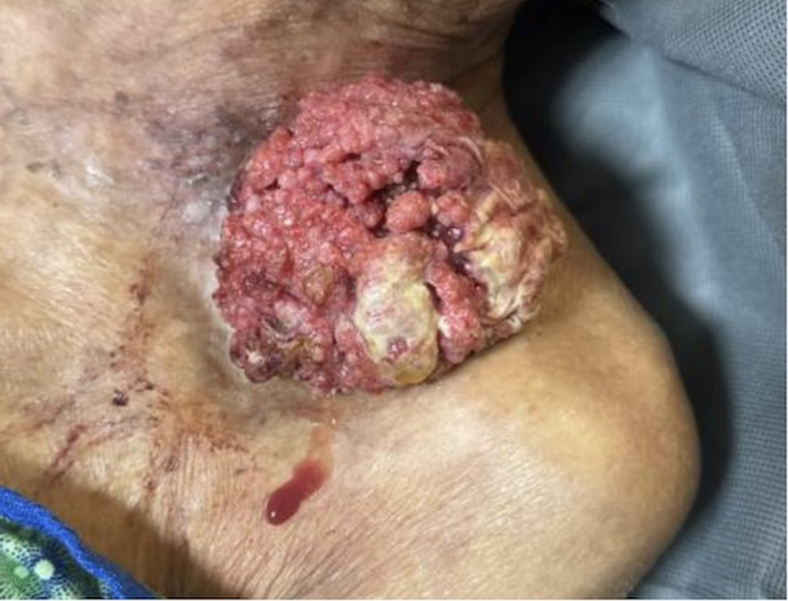
Large squamous cell carcinoma on the left chest. The mass had been growing for the past year with no medical evaluation.

**Figure 2:**
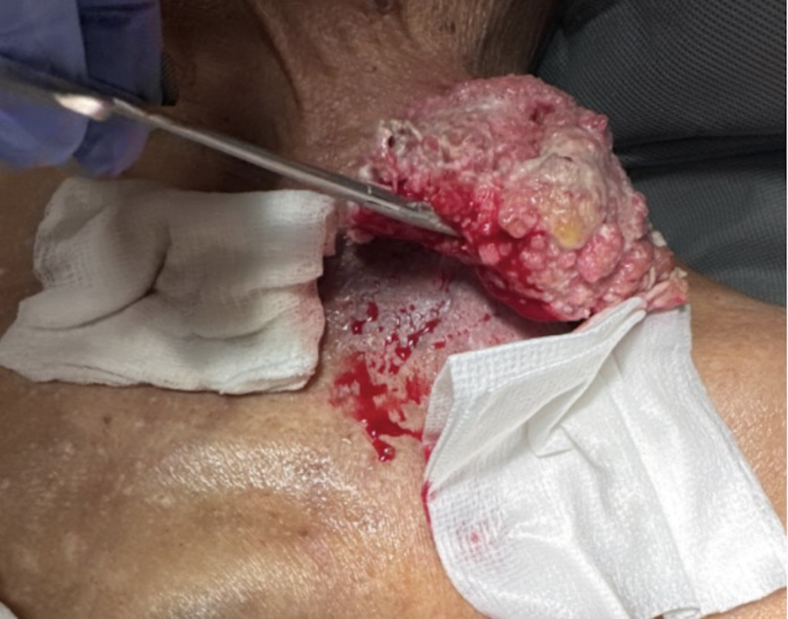
Debridement of squamous cell carcinoma. The image reveals the fungating mass to be superficial and attached by a stalk to his chest wall. Hydrogen peroxide and rubbing alcohol were used to clean the wound.

A 92-year-old male presented to the emergency department with a large mass on his left chest that had been growing for the past year with no medical evaluation (Fig 1). On examination, the mass was found to be fungating with living, moving maggots growing off the side of his chest. There was no active bleeding noted. Hydrogen peroxide and rubbing alcohol were used to clean the wound. The fungating mass appeared to be superficial and attached by a stalk to his chest wall (Fig 2). He was put on intravenous (IV) antibiotics due to leukocytosis seen by a white blood cell count of 16. He underwent a computed tomography (CT) scan that suggested some sort of malignancy such as squamous cell carcinoma and was ultimately hospitalized for further medical evaluation.

## Diagnosis

### Squamous Cell Carcinoma

The diagnosis was made according to clinical presentation and CT scan. In the emergency room, a CT of the chest, abdomen, and pelvis was done to understand the severity of the carcinoma. CT scans are the most accurate modality to detect bony invasion and nodal metastasis for cancer.^1^ The patient had an ill-defined heterogeneous medial left supraclavicular mass closely associated with the thyroid gland with a second separate hypervascular 7.2 cm exophytic mass arising from the lateral subclavicular subcutaneous soft tissue. The CT did not show signs of generalized thoracic lymphadenopathy, lung nodules, or acute pulmonary process.

Squamous cell carcinoma is a life-threatening subtype of skin cancer that usually starts as a small, red, painless lump of the skin. It usually occurs on areas of the skin that have been repeatedly exposed to strong sunlight, such as the head and hands.^2^ The main way to diagnose squamous cell carcinoma is with a biopsy, which involves having a small piece of the tissue removed from the suspicious area and examined in a laboratory. This was done during the patient’s hospital admission for surgical consultation, debridement, and further evaluation.

Most squamous cell carcinomas (95%-98%) can be cured if treated early, but survival drops to below 50% at 5 years once the cancer spreads.^2^ Treatment options for localized disease include surgical removal, freezing with liquid nitrogen, radiation therapy, laser therapy, and medications.^2^ The patient is currently getting treated with immunotherapy treatments and wound care regularly by Home Health.

## Funding and Support

By *JACEP* *Open* policy, all authors are required to disclose any and all commercial, financial, and other relationships in any way related to the subject of this article as per ICMJE conflict of interest guidelines (see www.icmje.org). The authors have stated that no such relationships exist.

## Conflict of Interest

All authors have affirmed they have no conflicts of interest to declare.
